# Validation of the Finnish Translation of the Acceptance and Action Questionnaire‐II Among Finnish Mental Health Professionals

**DOI:** 10.1111/scs.70169

**Published:** 2025-12-29

**Authors:** Katariina Keinonen, Sasha Zabelski, Skyler D. Prowten, Vojna Tapola, Robert J. Cramer

**Affiliations:** ^1^ Department of Psychology University of Jyväskylä Jyväskylän yliopisto Finland; ^2^ Department of Epidemiology and Community Health University of North Carolina at Charlotte Charlotte North Carolina USA

**Keywords:** AAQ‐II, compassion fatigue, experiential avoidance, mental health providers, psychological flexibility, validity

## Abstract

**Introduction:**

Mental health providers frequently face challenging working conditions and professional stress related to exposure to clients' emotional suffering and traumatic experiences. These challenges contribute to high levels of compassion fatigue among providers and highlight the need to understand factors that may support mental health professionals' own wellbeing. One such factor may be psychological flexibility.**Methods:** The current study aimed to assess the reliability and validity of one measure of psychological flexibility, the Finnish translation of the Acceptance and Action Questionnaire‐II (AAQ‐II) among mental health providers and trainees. Utilising a cross‐sectional design, the study examined the factor structure and convergent validity with compassion fatigue and compassion satisfaction (ProQOL), as well as divergent validity with social desirability (SDRS‐5).

**Results:**

The results supported the use of the AAQ‐II‐FV as a one‐factor measure of psychological flexibility, showing excellent internal consistency. The AAQ‐II‐FV demonstrated significant convergent validity with compassion satisfaction and secondary traumatic stress, but not with burnout. Divergent validity was established with no significant correlation to social desirability.

**Discussion:**

These findings suggest that the AAQ‐II‐FV is a reliable measure of psychological flexibility among mental health providers and trainees.

## 1 Introduction

Mental health providers' work with clients who struggle with psychological wellbeing is challenging. Challenges include frequent exposure to intense emotional suffering, suicidal ideation, and recount of patients' traumatic life experiences [1]. On top of the challenges that mental health providers face in their day‐to‐day work, the mental health sector is under severe financial strain which has contributed to job instability and understaffing [2]. Similar difficulties are faced by those who provide mental health services as part of community and third sector services, such as non‐profit organisations and associations. Indeed, all the above‐mentioned challenges were consistently identified in a qualitative study with peer mental health providers [3]. In addition, providers frequently highlighted additional challenges like difficulty managing their own mental health, less than ideal working conditions (e.g., low pay), and insufficient training.

Engagement in this work can result in negative outcomes for providers' own wellbeing. Previous literature established that some of the processes that may contribute to these negative effects, include secondary traumatic stress and burnout that can lead to compassion fatigue [4, 5]. Secondary traumatic stress is defined by the experience of post‐traumatic stress symptoms caused by indirect exposure to trauma, that is, a client's personal account of a traumatic experience [6]. A systematic review of secondary traumatic stress among mental health providers found that they reported rates of secondary traumatic stress as high as 70% [7]. Further, mental health providers' prevalence of personal trauma was consistently associated with their development of secondary traumatic stress.

Secondary traumatic stress can also predispose mental health providers to burnout [8]. Provider burnout develops as a response to chronic exposure to professional emotional and interpersonal stress [9], which highlights the need to focus research attention on identifying possible buffers that can negate the effects of this exposure. Burnout can manifest as feelings of exhaustion (e.g., feelings of being overextended or drained), cynicism (e.g., detached response from work, dehumanisation), and professional inefficacy (e.g., decline in perceived professional competency and productivity; Lenz et al. 2024, [10]). Previous literature suggests that experiences of burnout are very common among mental health providers. Meta‐analyses suggesting pooled estimates of 40% of providers experiencing burnout [11], and some studies suggesting even higher rates with estimates of up to 70% of providers reporting high levels of burnout‐related symptoms [12].

The symptoms related to secondary traumatic stress and burnout can contribute to the development of compassion fatigue, a state of psychological or physical distress that develops as result of an ongoing, challenging relationship with demanding clientele [10, 13, 14]. For example, a recent study found that providers exposed to workplace violence reported significant higher compassion fatigue (as measured by the secondary traumatic stress and burnout subscales of the Professional Quality of Life Scale [PROQOL] [10]) relative to those who did not work in these settings [15]. The high prevalence of experiences that predispose mental health providers to psychological and emotional suffering requires attention in the training and supervision practices for health care professionals and particularly providers who work with high‐risk clients. To respond to this need, it is important to establish effective ways to increase the provider's behavioural repertoire in the face of their challenging work. Recent findings suggest that one potential approach to supporting provider wellbeing is increasing providers' psychological flexibility, that can act as a buffer against compassion fatigue [16].

### 1.1 Psychological Flexibility

Psychological flexibility refers to the ability and willingness to connect with the present moment's thoughts and feelings to pursue meaningful, valued actions that align with an individual's values [17, 18]. Psychological flexibility is a defining factor of Acceptance and Commitment Therapy (sometimes also Acceptance and Commitment Training, ACT), and is comprised of six intertwined processes: acceptance, defusion, present‐moment awareness, self‐as‐context, values, and committed action [18]. Acceptance refers to willingness to experience distress when this serves pursuing one's goals. Defusion refers to the ability to observe thoughts as separate from the things they describe. Present‐moment awareness reflects mindful contact with direct experience. Self‐as‐context is defined as differentiating between the self and inner experiences (i.e., thoughts and emotions). Values refer to individually chosen life directions that can guide goal‐setting. Committed action refers to the ability to persist in actions that serve valued ends even in the face of inevitable difficulty and distress [19].

Psychological flexibility has been shown to mediate the effect of stress and distress among various populations and contexts, including professionals working in the healthcare field [16]. As providers often report frequent exposure to traumatic experiences, emotional suffering and suicidal ideation [1], adequate sufficient self‐care practices can be very important for these professionals. Research on interventions and training programs for professionals of both general and mental health suggest providers can significantly benefit from acceptance and mindfulness‐based interventions. For example, Mindfulness‐Based Stress‐Reduction (MBSR) has been shown to reduce stress and burnout and improve self‐compassion and mindfulness among providers [20]. However, while ACT‐based trainings and intervention programs have been effective in reducing stress and improving psychological flexibility and mindfulness, evidence on effectiveness for burnout, self‐compassion and psychological wellbeing among mental health professionals has not been consistent [21], suggesting that further research is needed to understand the relationship between psychological flexibility and domains of wellbeing among providers. For these reasons, it is important to accurately measure psychological flexibility among mental health providers to aid in the understanding and development of ACT strategies with the hopes of alleviating emotional strain, improving health and wellbeing, and ensuring patient safety.

### 1.2 Acceptance and Action Questionnaire‐II: Development and Use Considerations

One of the most frequently used measures of psychological flexibility is the Acceptance and Action Questionnaire (AAQ‐II [22]). It is a brief 7‐item scale that was originally designed to be used across diverse populations as a measure of general psychological (in)flexibility. The AAQ‐II has been translated to at least 23 languages and it has demonstrated adequate psychometric properties in various clinical and non‐clinical samples [23]. The exact wording of the items of the AAQ‐II is mostly focused on experiential avoidance, a specific domain of psychological inflexibility, and critical views have been raised regarding the use of the AAQ‐II as a general measure of psychological flexibility (e.g., [24] and [25]). Despite these concerns, the AAQ‐II has been shown to predict various mental health problems and psychological symptoms [26]. Previous research also established that the AAQ‐II mediates outcomes in psychological treatments based on ACT, which aims to increase psychological flexibility to alleviate psychological illbeing and improve wellbeing (e.g., [27]). These findings support the hypothesis that the AAQ‐II can measure processes that act as buffers or risk factors for psychological health and that should be targeted in interventions to support wellbeing.

In addition to the general AAQ‐II, several symptom‐ or population‐specific versions of the AAQ‐II have developed to improve sensitivity in detecting psychological flexibility in a particular domain of interest, such as psychological flexibility related to chronic pain [28], diabetes [29], obsessions and compulsions [30], and parenting [31], among others. These context‐specific versions of the AAQ‐II have mostly been examined in relation the original scale, and there is considerable variation in the extent to which psychometric properties of each version was examined. While some of the context‐specific scales have outperformed the generic approach of the original AAQ‐II, reviews of the validity and utility of these versions still recommend using both general and domain‐specific scales where possible to offer more reliable findings [23].

While commonly used also in research among Finnish populations, the Finnish translation of the AAQ‐II has not been formally validated, and the psychometric properties of the Finnish scale have not been thoroughly examined. Previous literature using the AAQ‐II suggests excellent reliability across diverse populations, including participants experiencing persistent physical symptoms (Cronbach's *α* = 0.92 [32]), elderly caregivers (Cronbach's *α* = 0.91 [33]) and university students seeking psychological support (Cronbach's *α* = 0.87 [34]), among other populations. However, to the best of the authors' knowledge, a more detailed examination of the psychometric properties of the Finnish AAQ‐II translation have not been previously reported.

### 1.3 The Present Study

The current study utilised a cross‐sectional design to examine the psychometric properties of the Finnish translation for the Acceptance and Action Questionnaire‐II (AAQ‐II [22]) among a sample of Finnish mental health providers and trainees. The aim was to (1) examine the factor structure of the scale, and (2) examine if the scale will show convergent validity with compassion fatigue and divergent validity with social desirability. The following hypothesis (H) were tested to establish these aims:

*The factor structure of the AAQ‐II‐FV will support the use of the scale as a one‐factor measure of psychological flexibility*.

*The AAQ‐II‐FV will show convergent validity with compassion fatigue measured through the ProQOL scale*.

*The AAQ‐II‐FV will show divergent validity with social desirability measure through the SDRS‐*5 *scale*.

## 2 Method

### 2.1 Design

The current study is a part of a larger research project focused on developing and validating new tools to evaluate the professional competencies of suicide prevention among Finnish mental health providers that was carried out in Finland between January 2024 and November 2024. The study protocol and materials were approved by the Institutional Review Board of UNC Charlotte. The study used a cross‐sectional design.

### 2.2 Recruitment

Participation required filling out an anonymous online survey. Informed consent was obtained from all participants. The anonymous survey was distributed using invitation letters shared via online communication channels (e.g., email lists, newsletters, web pages and social media communications) of several mental health organisations, and training and research institutions across Finland. Partnering institutions were asked to share the invitation with their employees and/or trainees. Additionally, members of each agency were encouraged to share the invitation with collaborators who met the study criteria. The invitation letter provided participants with preliminary information of the study and directed interested participants to the online survey link that included the full informed consent information letter. The link to the survey and information letter was also distributed in training events that were organised in collaboration with the partnering institutions.

### 2.3 Participants

To participate in the current study, participants had to meet the following inclusion criteria: (1) 18 years of age or older; (2) living in Finland; and (3) working as a licenced mental health services practitioner or currently a trainee to become a licenced mental health services practitioner. A mental health services practitioner was defined as current work or training in any of the following disciplines: nurse, psychologist, psychotherapist, psychiatrist or medical doctor, social worker, occupational therapist, other licenced health care professional, and other licenced social welfare professionals.

The full sample demographic information is summarised in Table 1. Participants were practising mental health clinicians and trainees living in Finland (*N* = 101). Participants were mostly middle‐to‐older age (34.7%) and women (83.2%). The primary territory of practice for participants was either in the Helsinki‐Uusimaa region (38.6%) or the Northeastern Finnish region (31.7%). Most clinicians practised in public healthcare settings (52.5%) in predominantly either a psychiatric specialist office (35.6%) or other type of office (41.6%). Participants were primarily licensed as a nurse (40.6%), psychologist (19.8%) or were unlicensed (i.e., trainee participants or professionals who work outside of healthcare services; 16.8%). Most participants were not currently in training (64.4%). There was a wide range of years of clinical experience.

**TABLE 1:** Participant characteristics.

Characteristic	*n*	%	M	SD
Age				
Less than 25	15	14.90		
26–35	12	11.90		
36–45	26	25.70		
46–55	35	34.70		
56–65	12	11.90		
65 and older	1	1.00		
Gender				
Woman	84	83.20		
Man	15	14.90		
Other	1	1.00		
Prefer not to answer	1	1.00		
Region				
Helsinki	39	38.60		
Southern Finland	13	12.90		
Western Finland	16	15.80		
Northeastern Finland	32	31.70		
Åland	0	0		
Work setting				
General healthcare	7	6.90		
Occupational healthcare	6	5.90		
College	4	4.00		
Prison	5	5.00		
Psychiatry	36	35.60		
Other	42	41.60		
Licensure				
Nursing	41	40.60		
Psychologist	20	19.80		
Psychotherapist	15	14.90		
Psychiatrist or doctor	5	5.00		
Social worker	2	2.00		
Occupational therapist	2	2.00		
Other healthcare professional	9	8.90		
Other social work professional	12	11.90		
No licensure	17	16.80		
Training				
Currently in psychotherapist training	13	12.90		
Currently studying psychology	17	16.80		
Other mental health professional training	6	5.90		
Currently in social work training	2	2.00		
Not currently in training	65	64.4		
Years of clinical experience				
0–5	34	33.70		
6–10	15	14.90		
11–15	15	14.90		
16–20	12	11.90		
More than 20	24	23.80		
Measures				
AAQ‐II‐FV			29.75	3.52
SDRS‐5			0.82	1.02
ProQOL—Compassion			38.00	5.82
ProQOL—Burnout			29.75	3.52
ProQOL—Secondary Traumatic Stress			18.58	4.77

Abbreviations: AAQ‐II‐FV, Acceptance and Action Questionnaire II – Finnish Version; M, Mean; *n*, subgroup sample size; ProQOL‐Burnout, Professional Quality of Life‐Burnout Subscale; ProQOL‐Compassion, Professional Quality of Compassion Satisfaction Subscale; ProQOL‐Secondary Traumatic Stress, Professional Quality of Life‐Secondary Traumatic Stress Subscale; SD, Standard deviaition; SDRS‐5, Socially Desirable Response Set Five Item Survey.

### 2.4 Translation

For the AAQ‐II‐FV, the Finnish translation from 2007 that is widely available and used in existing Finnish research samples was used as the translation for the current analyses. For all other scales, we followed the translation process described below. The translation process was designed to follow the recommendations regarding best practices for translation of surveys into new languages (e.g., [35]). In essence, a translation/back translation process was combined with team‐based and expert review procedures. First, the first author translated the surveys from English to Finnish. Next, a doctoral researcher fluent in both English and Finnish translated the surveys back from Finnish to English independent from the initial translation. Two other independent reviewers then revised the backtranslation to ensure precision. Finally, the original translation and the backtranslation were reviewed by the first and last author to discuss culturally appropriate phrasing where needed. All discrepancies and alterations to item wording were discussed in‐depth with the last author to resolve questions.

### 2.5 Survey and Data Collection Procedure

Data was collected via Qualtrics survey software. The survey contained: e‐consent, eligibility screening questions, and the primary survey. Primary survey measures were randomised to account for order effects. All participants were asked to familiarise themselves with the informed consent information letter that was included in the first page of the survey and give their consent if they were willing. Participants had the opportunity to contact the research team if they had any further questions before consenting. After consenting, participants were presented with the eligibility screening questions. Then, the survey continued to demographic questions, and self‐report scales of key outcome measures. Participants were able to skip items in the scales if they did not wish to answer. Upon survey completion, participants were debriefed using a written message that was included in the online survey. The debriefing included contact information and information for mental health provider locators and publicly available crisis support resources in Finland in case the survey had caused any emotional distress. Participants were also provided with contact information of the research team. Participants were encouraged to save the information provided in the debriefing.

### 2.6 Measures

#### 2.6.1 Demographics

Participants were asked to provide the following demographic information: age, gender, employment type, primary work setting, educational level, licensure status, trainee status, state of residence, years of clinical experience, and prior suicide prevention training. Demographic information was collected using the Finnish Social Sciences Data Archives [36] recommendations on minimising personal data collection and ensuring anonymity by classifying demographic data in a way that would not allow identification of individual participants directly or by combining different types of information.

#### 2.6.2 Acceptance and Action Questionnaire‐II (AAQ‐II)

The AAQ‐II [22] questionnaire was used as a measure of self‐reported psychological flexibility. For the purposes of the current study, we interpreted the total score to reflect psychological inflexibility, particularly experiential avoidance. Participants were asked to rate their endorsement of seven items on a 7‐point Likert scale (1 = never true; 7 = always true). Items were summed to drive a total score, with higher scores indicating higher levels of experiential avoidance.

#### 2.6.3 Professional Quality of Life: Compassion Satisfaction and Compassion Fatigue (ProQOL)

Compassion fatigue and compassion satisfaction were measured using the ProQOL [10]. The 30‐item scale measured three aspects of professional quality of life, including compassion satisfaction (10 items), burnout (10 items) and secondary traumatic stress (10 items). Scores on the burnout and secondary traumatic stress subscales were used to indicate compassion fatigue levels, where higher scores indicate greater compassion fatigue. Responses were rated on a five‐point Likert scale (1 = never; 5 = very often). Items in each subscale (e.g., compassion satisfaction, burnout, and secondary traumatic stress) were summed to create sum scores. Scores of 22 or less were considered low, between 23 and 41 were considered moderate, and 42 or more were considered high. Each subscale of the ProQOL was found to have acceptable reliability in the current sample (Compassion Satisfaction Cronbach's *α* = 0.88; Burnout Cronbach's *α* = 0.75; Secondary Traumatic Stress Cronbach's *α* = 0.80).

#### 2.6.4 Socially Desirable Response Set‐5 (SDRS‐5)

The five item, Socially Desirable Response Set (SDRS‐5 [37]) was used to measure overly favourable self‐presentation styles in responding. Participants were asked to indicate the degree to which each statement described them on a 5‐point Likert scale (1 = definitely true; 5 = definitely false). Items were then binary coded (0 = not socially desirable; 1 = socially desirable) and summed for a total score. Higher scores indicated higher levels of presenting in a socially desirable way. Kuder–Richardson 20 (KR20) formula [38] was used to calculate binary outcome reliability. Estimated reliability for the SDRS‐5 in this study was poor (KR20 = 0.46).

## 3 Data Analysis

A total of 125 persons opened the survey link. We used several techniques to ensure data quality in online survey studies in line with guidelines in the methods literature (e.g., [39]). First, responses with less than 60% of the survey completed were removed (*n* = 15). Additionally, after determining percentile values for time‐to‐completion for each survey respondent, any outliers beyond the 5th or 75th response time percentiles were dropped (*n* = 9) leaving a final sample size of 101. This sample size was deemed sufficient to move forward with testing the AAQ‐II‐FV factor structure; sample sizes of less than 100 are required for analysis of single‐factor structures with 8 or less items and moderate‐to‐large expected factor loadings [40]. Missingness on survey items ranged from 0.0% to 8.9%. To account for missingness, regression‐based multiple imputation [41] was performed with the following parameters: 20 imputations, 2000 case draws, and the AAQ‐II‐FV items serving as predictors to supplant responses for all items of interest.

To assess H1, we conducted confirmatory factor analysis (CFA) using AMOS v. 24 [42]. Specifically, we tested a single‐factor structure model of the original AAQ‐II [22]. Maximum likelihood estimation was used for normally distributed continuous data [43]. Model fit was evaluated using the following set of fit statistics: Chi‐square test, Tucker‐Lewis Index (TLI), Comparative Fit Index (CFI), Root Mean Square Error of Approximation (RMSEA) and Root Mean Square Residual (RMR). Minimally acceptable guidelines for acceptable fit were as follows: TLI > 0.90, CFI > 0.90, RMSEA < 0.08, SRMR < 0.08, and non‐significant Chi‐square value [44]. However, gradations of fit (e.g., inadequate vs. acceptable vs. good/excellent) have been articulated in the literature, with combinations of more conservative fit statistics guidelines representing better model fit to the data. Cronbach's *α* was used to assess internal consistency. Bivariate correlations were run to examine convergent validity with the ProQOL subscales (H2) and divergent validity with the SDRS‐5 (H3).

## 4 Results

### 4.1 H1: Factor Structure and Reliability of the AAQ‐II‐FV

The first hypothesis was that the AAQ‐II‐FV factor structure would support the use of the scale as a one‐factor measure of psychological inflexibility. This hypothesis was primarily supported. The AAQ‐II‐FV single factor structure showed marginal‐to‐adequate fit to the data, χ^2^(101) = 37.62, *p* = 0.001; CFI = 0.95, TLI = 0.90, RMSEA = 0.13 (90% CI = 0.08, 0.18), SRMR = 0.04. All items loaded significantly and in the expected positive direction (λ **=** 0.69–0.85, all *p*s < 0.001). The scale demonstrated excellent internal consistency (*α* = 0.92).

### 4.2 H2: AAQ‐II‐FV Convergent Validity With ProQOL Subscales of Compassion Fatigue (Burnout, Traumatic Stress) and Compassion Satisfaction

The second hypothesis was that the AAQ‐II‐FV would show convergent validity with compassion fatigue as measured with the ProQOL. This hypothesis was partially supported. The AAQ‐II‐FV was significantly and negatively correlated with compassion satisfaction (*r* = −0.57, *p* < 0.001). This pattern was expected because higher AAQ‐II‐FV scores reflect lower psychological flexibility. For the factors of compassion fatigue that were secondary traumatic stress and burnout, the AAQ‐II‐FV was significantly and positively correlated with secondary traumatic stress (*r* = 0.42, *p* < 0.001). Contrary to expectations, burnout showed no relationship with the AAQ‐II‐FV (*r* = −0.03, *p* = 0.75). Significant correlations for two out of three subscales showed evidence of convergent validity.

### 4.3 H3. AAQ‐II‐FV Divergent Validity With the SDRS‐5

The third hypothesis was that the AAQ‐II‐FV would show divergent validity with socially desirable response style. H3 was supported. Social desirability showed no relationship to the AAQ‐II‐FV (*r* = −0.06, *p* = 0.52).

## 5 Discussion

The present study assessed the reliability and construct validity of the AAQ‐II among a sample of Finnish mental health providers and trainees. Specifically, we tested hypothesis focused on the reliability of AAQ‐II as a measure of psychological flexibility among Finnish mental health providers and trainees, and factor analytic solutions of the AAQ‐II in this population: (1) The factor structure of the Finnish translation of the AAQ‐II would support the use of this scale as a one‐factor measure of psychological inflexibility, (2) The scale would demonstrate convergent validity with compassion satisfaction and compassion fatigue (the burnout and secondary traumatic stress subscales of the PROQOL), and (3) the scale would demonstrate divergent validity with social desirability measured by the SDRS‐5. Among the current sample of Finnish mental health providers and trainees, the AAQ‐II‐FV demonstrated excellent reliability as a measure of psychological inflexibility. Furthermore, in support of H1, our factor analytic solution supports the use of the AAQ‐II as a one‐factor measure of psychological inflexibility.

The current findings on the excellent reliability and support one‐factor structure are in line with findings regarding other language versions in European languages (e.g., [45, 46, 47]). The universality of the construct of psychological inflexibility as measured by the AAQ‐II has also been supported in explicit examination of cultural variation across European languages [48]. As the most widely used measure of psychological inflexibility, the AAQ‐II has consistently demonstrated adequate psychometric properties [23]. The current results add to this literature by showing good psychometric properties among Finnish mental health providers and trainees alike.

In support of H2, increased levels of psychological flexibility were associated with greater levels of compassion satisfaction and lower levels of secondary traumatic stress. However, there was a non‐significant association between psychological flexibility and burnout. The ProQOL has been used in Finnish samples before, but the scale has not been formally validated. In fact, concerns for the psychometric properties and validity of the ProQOL, particularly for the burnout subscale, among healthcare populations have been raised (e.g., [49]). Indeed, the internal consistency for the burnout subscale was worse when compared to the compassion satisfaction and secondary traumatic stress subscales. Previous studies have used both bifactorial scoring of compassion satisfaction and compassion fatigue [50] and three‐factor scoring (i.e., compassion satisfaction, burnout and secondary traumatic stress [51]). In the current study, the ProQOL was translated to Finnish following recommendations for best practices (e.g., [35]) to ensure translation quality. The ProQOL was then used to assess convergent validity and the three‐factor scoring was utilised in analyses. However, while the AAQ‐II‐FV showed convergent validity with the compassion satisfaction and secondary traumatic stress subscales as expected, no association was established with the burnout scale. It is possible that this is due to the lower reliability of the burnout scale. The current results indirectly support the notion that the burnout subscale may not perform optimally among samples that consist of healthcare providers.

Finally, in support of H3, there was non‐association observed between psychological flexibility and social desirability. This finding suggests the overall degree of positive impression management was low in this sample, and unrelated to responses to the AAQ‐II. This finding supports the assumption that the AAQ‐II can be used as a tool to measure psychological flexibility among mental health providers in the contexts of research focused on provider wellbeing on training program evaluation and provider skill development.

Overall, our findings suggest that the AAQ‐II is a reliable measure of psychological flexibility among Finnish mental health providers and trainees. While efforts to develop more sensitive and varied scales to capture processes of psychological flexibility continue, the AAQ‐II still offers a brief, reliable approach to measuring experiential avoidance, or psychological inflexibility in the face of unwanted inner experiences.

The present study possesses several limitations. First, the sample size was limited to 101 participants. While sufficient for the current analyses, the relatively small number of participants limits generalizability. Specifically, generalizability to ethnic and racial minorities is severely limited. Because Finland is largely racially homogenous, official statistics do not group citizens by ethnicity, and only report the proportion of population with foreign background at around 10% [52]. Given the small portion of racial and ethnic minorities, the anonymous survey design did not include race and ethnicity in the demographic data to prevent identification by combining demographic variables for individual participants. However, it should be noted that previous studies have found mixed results when testing for AAQ‐II measurement invariance between racial and ethnic groups [53, 54], though this has not been investigated within a Finnish cultural context. Future studies need to focus on the study of psychological flexibility among racial and ethnic minorities in Finland as there may be differences in understanding and expression of psychological flexibility.

In accordance with the European Union's General Data Protection Regulation (GDPR; Regulation 2016/679) [55], all demographic variables were measured categorically in the current design that utilised an anonymous survey. However, collection of all demographic data as categorical precludes analysis of continuous demographic variables such as participant age. As a result, our findings must be interpreted with caution before application to any particular age group of Finnish mental health providers.

Finally, it should be noted that while we took care to follow best practices in survey translation [35], there is still the potential for lost meaning in the process. As psychological flexibility is a latent construct, and cannot be overtly observed, our translation depends on a shared cross‐cultural understanding of the construct. Additionally, not all authors on the research team were fluent in Finnish at the time of data collection, resulting in our team's dependence on cross‐cultural communication and understanding of psychological flexibility.

Mental health providers face chronic exposure to professional emotional and interpersonal stress. It is important to devote research efforts in establishing potential protective factors or buffers that can mitigate the effects of work‐related negative effects to wellbeing. Psychological flexibility can be one of the factors that should be further explored as a trainable set of processes that support psychological health. If the role of psychological flexibility for protecting mental health professionals' wellbeing from adverse effects of emotionally demanding work is further corroborated in future research, training programs could integrate modules focused on building resilience through teaching psychological flexibility skills. Psychological flexibility may have a particularly important role as a possible buffer for compassion fatigue when working with high‐risk patients and trauma. Again, if this conclusion is further supported in future research, training practices could be developed to foster psychological flexibility as part of practitioner‐focused training content (e.g., self‐care practices and debriefing). Addressing provider psychological flexibility in mental health professionals' training is also supported by previous studies that suggest psychological flexibility may result in better outcomes for both mental health providers and their clients by improving providers' ability to adopt an open and aware stance towards the here and now experience when working with trauma [56]. The AAQ‐II offers a reliable instrument to measure psychological flexibility in future research among mental health providers and trainees.

## Author Contributions

**Katariina Keinonen:** conceptualization, writing – original draft, writing – review and editing. **Sasha Zabelski:** formal analysis, methodology, writing – original draft, writing – review and editing. **Skyler D. Prowten:** data curation, methodology, administration, resources, writing – original draft, writing – review and editing. **Vojna Tapola:** writing – review and editing. **Robert J. Cramer:** conceptualization, data curation, resources, supervision, writing – original draft, writing – review and editing.

## Funding

This project was funded in part by the University of Jyväskylä Visiting Fellow Programme (2024).

## Ethics Statement

The study protocol and materials were approved by the Institutional Review Board of UNC Charlotte.

## Conflicts of Interest

The authors declare no conflicts of interest.

## Data Availability

Data statement: Data from this project are protected by European law, and consenting processes that preclude secondary access to subject data.
